# Radioiodinated Tau Imaging Agent III Molecular Modeling, Synthesis, and Evaluation of a New Tau Imaging Agent, [^125^I]ISAS in Post-Mortem Human Alzheimer’s Disease Brain

**DOI:** 10.3390/molecules29143308

**Published:** 2024-07-13

**Authors:** Stephanie A. Sison, Cayz G. Paclibar, Christopher Liang, Jogeshwar Mukherjee

**Affiliations:** Preclinical Imaging, Department of Radiological Sciences, University of California-Irvine, Irvine, CA 92697, USA; sisonsa@uci.edu (S.A.S.); cgpaclib@uci.edu (C.G.P.); liangc@uci.edu (C.L.)

**Keywords:** [^125^I]ISAS, [^125^I]INFT, [^125^I]IPPI, post-mortem human Tau, Alzheimer’s disease, neurofibrillary tangles, autoradiography

## Abstract

Using a molecular modeling approach for Tau-binding sites, we modified our previously reported imaging agent, [^125^I]INFT, for the potential improvement of binding properties to Tau in an Alzheimer’s disease (AD) brain. Two new derivatives, namely [^125^I]ISAS and [^125^I]NIPZ, were designed, where binding energies at site 1 of Tau were −7.4 and −6.0 kcal/mole, respectively, compared to [^125^I]INFT (−7.6 kcal/mole). The radiosynthesis of [^125^I]ISAS and [^125^I]NIPZ was carried out by using iodine-125 and purified chromatographically to achieve >90% purity. In vitro binding affinities (IC_50_) for Tau were as follows: INFT = 7.3 × 10^−8^ M; ISAS = 4.7 × 10^−8^ M; NIPZ > 10^−6^ M. The binding of [^125^I]ISAS to gray matter (GM) correlated with the presence of Tau in the AD brain, confirmed by anti-Tau immunohistochemistry. [^125^I]NIPZ did not bind to Tau, with similar levels of binding observed in GM and white matter (WM). Four radiotracers were compared and the rank order of binding to Tau was found to be [^125^I]IPPI > [^125^I]INFT > [^125^I]ISAS >>> [^125^I]NIPZ with GM/WM ratios of [^125^I]IPPI = 7.74 > [^125^I]INFT = 4.86 > [^125^I]ISAS = 3.62 >> [^125^I]NIPZ = 1.24. The predictive value of Chimera–AutoDock for structurally related compounds binding to the Tau binding sites (measured as binding energy) was good. A binding energy of less than −7 kcal/mole is necessary and less than −8 kcal/mole will be more suitable for developing imaging agents.

## 1. Introduction

Alzheimer’s disease (AD) is the most common type of dementia, accounting for 50 to 80 percent of dementia cases characterized by the accumulation of amyloid β (Aβ) plaques and neurofibrillary tangles (NFT) in the brain (and resulting in a huge cost to society) [1,2]. Molecular biomarkers for AD are now indispensable for the clinical definition of the process and stage of the disease [3]. There is now increased focus on Tau, as a more accurate, early predictive marker for AD diagnosis. Phosphorylated p-tau immunoassays, p-tau181 [4,5], and p-tau217 [6] confirmed that plasma p-tau has high sensitivity and specificity to detect AD neuropathology [7,8]. Studies suggest that p-tau can quantify longitudinal changes in tau pathology, identify neurodegeneration, and predict AD progression [9]. By using the antibodies AT100 and pS396, phospho-tau immunostaining in neurons from the hippocampal CA1 region with tau pathology indicates that the AT100/pS396 ratio decreases in CA1 in accordance with the severity of the disease and has been suggested as a measure of disease progression [10]. Higher correlations between p-tau217 and [^18^F]flortaucipir were corroborated [7].

Alongside progress in Aβ imaging, efforts are underway on the development and use of NFT PET imaging agents [11]. Consistent with CSF measures of p-tau, current data suggest that in addition to Aβ imaging, NFT imaging can play an essential role in clinical studies for the evaluation of disease progression [12]. Direct measures of brain biomarkers for earlier diagnosis need to be pursued in order to understand the stage of the disease. One of the earliest PET radiotracers was [^18^F]FDDNP, which was less selective for NFT with higher levels of nonspecific binding [13]. More selective PET radiotracers, such as [^18^F]THK5351, provide useful information on AD patients but have recently been reported to have significant off-target binding to monoamine oxidase (MAO B), reducing their potential use in AD diagnosis [14]. Pyrrole derivatives such as [^18^F]flortaucipir also appear to show some off-target MAO binding [15]. Nevertheless, studies using [^18^F]flortaucipir and the related [^18^F]RO6958948 continue for NFT imaging in AD [16]. Second-generation NFT PET radiotracers based on the azaindole structure, such as [^18^F]MK-6240, have been developed; they bind more selectively to the Tau protein. Off-target binding to MAO using in vivo may not be a concern [17]. They have high affinity and are capable of crossing the blood–brain barrier to be valuable for the application of in vivo NFT imaging in PET studies. As an NFT radiotracer, [^18^F]MK-6240 shows characteristic changes in the brains of patients with AD [18].

Currently, there are no radioiodine-based SPECT imaging agents in human studies for Tau and NFT. Recently, the radioiodinated derivative [^125^I]pyridoimidazopyridine has been developed for in vitro studies on NFT by one research group [19]. There are no further reports on its selectivity for Tau versus MAO off-target binding. The availability of SPECT imaging agents will allow nuclear medicine clinics, which may not have PET capability, to purchase the iodine-123-labeled radiopharmaceuticals for imaging studies. Also, the availability of iodine-124-labeled radiopharmaceuticals will allow flexible transportation to remote PET sites. Iodine-124—because of its longer half-life—will allow for extended imaging times, enable greater clearance of nonspecific binding and, thus, provide potentially better image contrast, which may be useful when small levels of NFT are present. Substituents at the 6-position of the isoquinoline ring seem to have only a small effect on the binding affinity to Tau. The availability of an iodine-125 azaindole derivative for in vitro studies of NFT will be valuable to complement studies with [^18^F]MK-6240. Therefore, we developed the [^125^I]IPPI derivative for Tau imaging, which is selective and lacks off-target binding in vitro [20]. There is a need for Tau imaging agents that can more reliably be useful below Braak IV stages of AD [21], discriminate between 3R and 4R Tau isoforms, have reduced off-target binding (such as binding to meninges [22]), and be useful for imaging other tauopathies [23]. Therefore, new derivatives, with lower lipophilicity and high binding to Tau, capable of differentiating between 3R and 4R, are needed.

Tau isoforms 3R and 4R are present in Alzheimer’s disease (AD, 3/4R), Pick’s disease (PiD, 3R), corticobasal degeneration (CBD, 4R), and progressive supranuclear palsy (PSP, 4R). Selective imaging agents for 3R and 4R will help the development of differential diagnostic methods for various tauopathies. Recent preliminary reports have suggested some Tau PET radiotracers bind to other tauopathies with varying degrees [23]. There is much discussion on the time course of the prevalence of 3R and 4R in AD and the differences in their relative toxicities in human AD [24].

**Figure 1:**
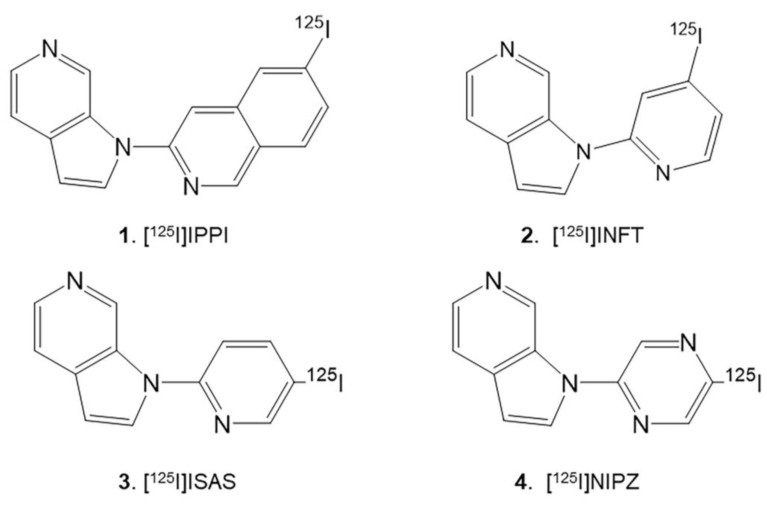
Radioiodinated tau binding radioligands under development: [^125^I]IPPI **1** [20]; [^125^I]INFT **2 [26]**; [^125^I]ISAS **3** (reported here); [^125^I]NIPZ **4** (reported here).

Results with [^125^I]IPPI (Figure 1 (**1**)) demonstrated the presence of Tau in the human AD postmortem brain selectively [20]. An iodine-124 analog, [^124^I]IPPI, was developed for in vivo PET imaging and [^125^I]IPPI was sensitive to different levels of Tau in postmortem human AD brain slices [25]. The lipophilicity (logP) of IPPI was found to be 4.34. For optimal in vivo imaging, a logP of approximately 2–2.5 is preferred to ensure optimal brain uptake across the blood–brain barrier and minimize nonspecific binding. In order to reduce the lipophilicity (logP) of [^125^I]IPPI, the quinoline ring was replaced with a pyridine ring to provide [^125^I]INFT (Figure 1 (**2**)) [26]. The logP of INFT was 2.96, significantly lower compared to IPPI. The binding of [^125^I]INFT to postmortem human AD brain slices was significant, but specific binding ratios of [^125^I]INFT were weaker compared to [^125^I]IPPI [26].

As previously discussed, the azaindole class of compounds has shown tolerance to a variety of substitutions in the isoquinoline ring [20]. Our goal was to assess if the pyridine ring would also be amenable to such substitutions. Thus, we investigated moving the position of the iodine atom in INFT as well as the addition of additional nitrogen in the ring (converting the pyridine ring to a pyrazine ring). These attributes can potentially provide improved binding affinities and lower lipophilicity, thus improving the properties of the imaging agent. Therefore, we prepared 5-iodo-2-(1H-pyrrolo[2,3-c]pyridine-1-yl)pyridine ([^125^I]ISAS; Figure 1, **3**) and 5-iodo-2-(1H-pyrrolo[2,3-c]pyridine-1-yl)pyrazine ([^125^I]NIPZ; Figure 1, **4**). Here, we report: (1) molecular docking studies of ISAS and NIPZ to human Tau; (2) assessment of logP; (3) synthesis of ISAS, NIPZ, and analogs; (4) measurement of binding affinity of the analogs for Tau using the postmortem AD brain; (5) radiosynthesis of [^125^I]ISAS and [^125^I]NIPZ; (6) autoradiographic studies of [^125^I]ISAS and [^125^I]NIPZ in postmortem AD brain slices.

## 2. Results

**Figure 2:**
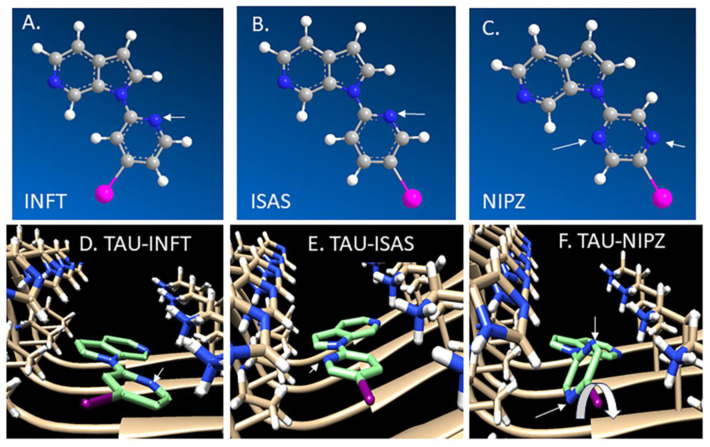
Tau filament (PDB 503L) molecular models: energy-minimized chemical structures of azaindole derivatives, INFT (**A**), ISAS (**B**), and NIPZ (**C**) used in auto-docking studies. (**D**–**F**) Chimera AD Tau model at site 1 showing binding of INFT (**D**; in green), ISAS (**E**; in green), and NIPZ (**F**; in green). In the cases of INFT and ISAS, the three nitrogen atoms (blue) are in the same plane, while in NIPZ, the two nitrogen atoms of the pyrazine ring are non-coplanar due to rotation (~90°) of the pyrazine ring (arrow in **F**). The iodine atom is seen in purple in all the panels.

Chimera–AutoDock was used to compare the binding of INFT, ISAS, and NIPZ (Figure 2) to the cryo-EM three-dimensional structure of Tau fibril [20,26,27]. For purposes of docking studies of INFT, ISAS, and NIPZ with the Tau model, energy-minimized molecular models were made using Chem Draw 3D (Figure 2A–C). Four sites in the Tau fibril were identified for IPPI binding [20]. Table 1 compares the binding energy values (Kcal/mol) at the four sites for IPPI, with the various pyridine derivatives reported here. Substitution of the isoquinoline ring of IPPI with a pyridine ring in INFT adversely affects binding energies in sites 2, 3, and 4. This was found to be the case for ISAS and NIPZ as well. Docking studies with INFT revealed that preferential binding at Site 1 (Figure 2D) was similar to the binding of IPPI at Site 1. The lack of the second phenyl ring in INFT (compared to isoquinoline in IPPI) potentially reduces the hydrophobic interactions, thus weakening the binding energies of sites 2–4. Compared to INFT, the binding energies of ISAS also bound similarly at Site 1 (Figure 2D,E) and were, thus, able to retain the low binding energy. This was not the case with NIPZ (Figure 2F). In the cases of INFT and ISAS (Figure 2D,E), the three nitrogen atoms (blue) are in the same plane, while in NIPZ, the two nitrogen atoms of the pyrazine ring are non-coplanar due to the rotation (~90°) of the pyrazine ring (arrow in Figure 2F). This non-coplanarity of NIPZ and its bromo analog, NBrPZ, seem to adversely affect the binding energies in all four sites, potentially weakening their binding affinity to Tau in all four sites.

Altering the position of the iodine atom in INFT (Figure 1 (**2**)) to ISAS (Figure 1 (**3**)) increased the lipophilicity from 2.96 to 3.18. As expected, the inclusion of the pyrazine ring in NIPZ (Figure 1 (**4**)) decreased the cLogP to 2.45. Replacing the iodine atom in NIPZ with bromine in NBrPZ further reduced the cLogP to 2.18. The lipophilicity of IPPI remained the highest at 4.34.

The binding affinity of the unlabeled compounds was evaluated autoradiographically in AD brain slices labeled with [^125^I]IPPI. The anterior cingulate of the subjects was first evaluated for the presence of Tau using [^125^I]IPPI; as expected, all AD subjects showed the presence of Tau, as reported previously [14]. Different compounds (Table 1) at concentrations of 10^−9^ M to 10^−5^ M were used for the competition assay with [^125^I]IPPI. Incubation of the brain slices with the respective compounds and [^125^I]IPPI was conducted in 30% alcohol in a PBS buffer using the reported procedures [14]. The binding of [^125^I]IPPI to Tau in brain slices was quantified in Digital Light Units (DLUs)/mm^2^ using the OptiQuant image analysis program. Data were analyzed using GraphPad Prism 9 software. Pyrazine derivatives, NIPZ and NBrPZ, did not exhibit significant binding to Tau. Binding affinities of ISAS and INFT were comparable, while IPPI had a marginally higher affinity.

**Figure 3:**
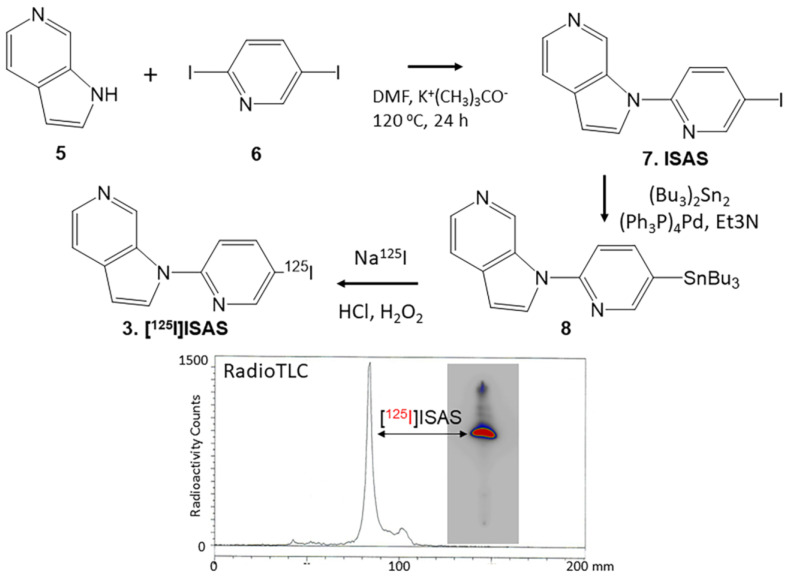
Synthesis of ISAS and [^125^I]ISAS: Azaindole **5** reacted with 2,4-diiodopyridine **6** in dimethylformamide (DMF) and potassium *tert*-butoxide (K^+^(CH_3_)_3_CO^−^ to provide ISAS **7**. INFT, **7** refluxed with bis(tributyltin) in the presence of tetrakis(triphenylphosphine)palladium(0) for 24 h to provide tributyltin precursor, **8**. Tributyl tin precursor **8** reacted with sodium [^125^I]iodide using hydrogen peroxide to provide [^125^I]ISAS, **3**. The radioactive thin layer chromatogram (RadioTLC) of [^125^I]ISAS shows a predominant peak (red) with a purity of >90%.

The synthesis of unlabeled ISAS **7** was carried out in a single step by reacting azaindole **5** with 2,4-diiodopyidine **6** (Figure 3). The nucleophilic displacement reaction resulted in displacing the iodine at the 2-position, resulting in ISAS, **7**. In order to prepare tributyltin derivative **8**, either ISAS or its bromo analog was used. Both precursors enabled an efficient exchange of the tributyl tin, which was purified and used for radiosynthesis.

The radioiodination of tributyltin derivative **8** was carried out using iodine-125 sodium iodide ([^125^I]NaI in sodium hydroxide solution; ARC Inc., St. Louis, MO, USA). Electrophilic substitution of the tributyltin derivative using hydrogen peroxide as the oxidant was carried out using our previously reported methods [16,19]. Tributyltin derivative **8** (0.1 mg in 0.1 mL ethanol) reacted with 3.7 MBq [^125^I]NaI and was allowed to proceed at room temperature for 60 min followed by termination using sodium bisulfite.

The purification and isolation of [^125^I]ISAS were carried out on preparative TLC. The product was extracted using dichloromethane and then dried using anhydrous MgSO_4_. RadioTLC confirmed a radiochemical purity of >90% [^125^I]ISAS with some minor impurities at a higher Rf (Figure 3). Using the molar activity of commercially available no-carrier-added [^125^I]sodium iodide, the molar activity of [^125^I]ISAS was estimated to be approximately 90 TBq/mmol. The radiochemical yield of [^125^I]ISAS was approximately 50%

**Figure 4:**
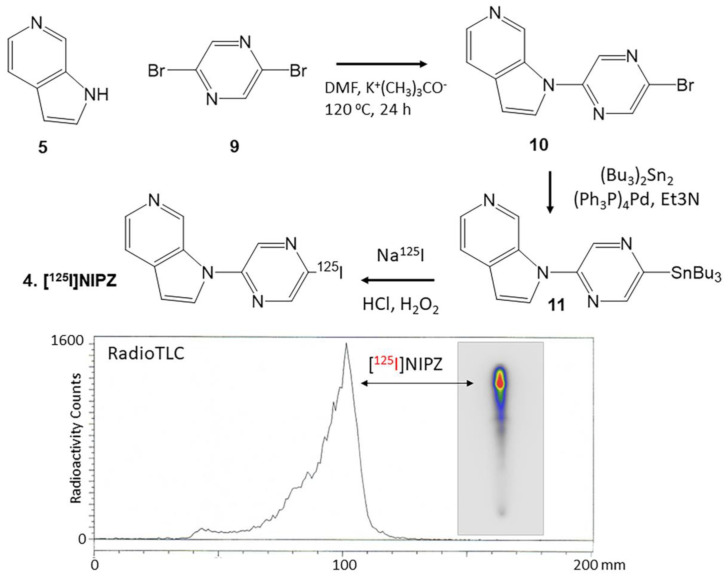
Synthesis of [^125^I]NIPZ: Azaindole **5** reacted with 2,5-dibromopyrazine **9** in dimethylformamide (DMF) and potassium *tert*-butoxide (K^+^(CH_3_)_3_CO^−^ to provide the brominated derivative, NBrPZ **10**. Refluxing the bromo derivative **10** with bis(tributyltin) in the presence of tetrakis(triphenylphosphine)palladium(0) for 24 h provided the tributyltin precursor, **11**. Tributyl tin precursor **11** reacted with sodium [^125^I]iodide using hydrogen peroxide to provide [^125^I]NIPZ, **4**. The radioactive thin layer chromatogram (RadioTLC) of [^125^I]NIPZ (red) shows a predominant peak with a purity of >90%.

The synthesis of unlabeled NBrPZ **10** was carried out in a single step by reacting azaindole **5** with 2,5-dibrompyrazine **9** (Figure 4). The nucleophilic displacement reaction resulted in displacing the bromine at the 2-position, resulting in NBrPZ, **10**. Tributyltin derivative **11** was prepared through an efficient exchange of the bromine with tributyltin, which was then purified and used for radiosynthesis.

The radioiodination of tributyltin derivative **11** was carried out using iodine-125 sodium iodide, similar to the previously described procedure. Tributyltin derivative **11** (0.1 mg in 0.1 mL ethanol) reacted with 3.7 MBq [^125^I]NaI and was allowed to proceed at room temperature for 60 min followed by termination using sodium bisulfite. The purification and isolation of [^125^I]NIPZ were carried out on preparative TLC as described previously for [^125^I]ISAS. RadioTLC confirmed a radiochemical purity of >90% [^125^I]NIPZ (Figure 4). Using the molar activity of the commercial no-carrier-added [^125^I]sodium iodide, the molar activity of [^125^I]NIPZ was estimated to be approximately 90 TBq/mmol. The radiochemical yield of [^125^I]NIPZ was approximately 30%.

**Figure 5:**
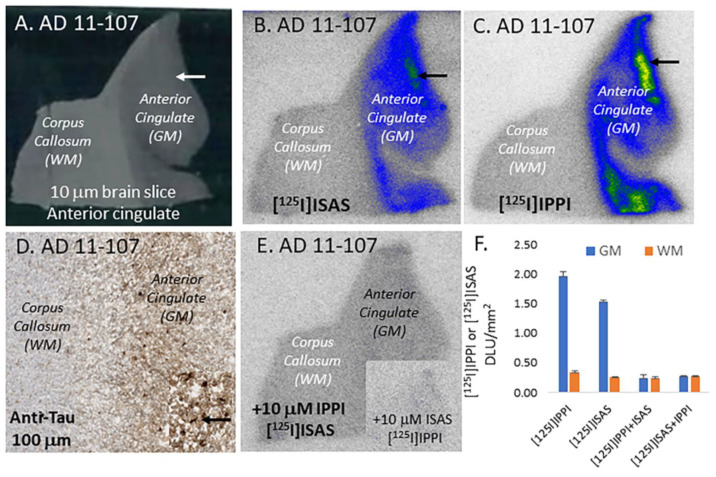
Postmortem human brain autoradiography [^125^I]ISAS: (**A**) human postmortem AD 11-107 brain anterior cingulate (10 μm thick sections) showing gray matter (GM) and white matter (WM). (**B**) Binding of [^125^I]ISAS to Tau (shown by arrows) in the gray matter regions of the anterior cingulate of AD 11-107 brain slice. (**C**) Binding of [^125^I]IPPI to Tau (shown by arrows) in the gray matter regions of the anterior cingulate of AD 11-107 brain slice. (**D**) Anti-Tau staining of AD 11-107 showing the presence of Tau in GM regions. Inset shows magnification (20 μm) of GM region shows Tau (arrow). (**E**) Displacement of [^125^I]ISAS from Tau binding sites by 10 μM IPPI (inset shows displacement of [^125^I]IPPI by 10 μM ISAS. (**F**) The plot of [^125^I]IPPI and [^125^I]ISAS binding to Tau in GM and reference region WM. The binding of [^125^I]IPPI in the GM was reduced in the presence of 10 μM ISAS and [^125^I]ISAS binding was reduced by 10 μM IPPI to levels of nonspecific binding in the WM. Ratios of GM/WM were: [^125^I]IPPI = 7.84; [^125^I]ISAS = 6.14; [^125^I]IPPI + 10 μM ISAS = 0.99; [^125^I]ISAS + 10 μM IPPI = 1.45.

The anterior cingulate from six AD subjects was used for the evaluation of [^125^I]ISAS binding. Figure 5 shows the binding of [^125^I]ISAS to one subject (AD 11-107) and a comparison with [^125^I]IPPI binding to the same subject. The brain slice (Figure 5A) consisted of the anterior cingulate (gray matter, GM) and corpus callosum (white matter, WM). Figure 5B shows the binding of [^125^I]ISAS to the GM regions in AD 11-107 with significantly lower binding in WM. A [^125^I]ISAS GM/WM ratio of 6.14 was measured. Similarly, an adjacent brain slice of AD 11-107 exhibited significant amounts of [^125^I]IPPI binding in the GM. A higher [^125^I]IPPI GM/WM ratio of 7.84 was measured, indicative of the better affinity of IPPI for Tau. The adjacent anti-Tau immunostained (IHC) brain section of AD 11-107 indicated the presence of abundant Tau (Figure 5D). Areas of Tau IHC in Figure 5D corresponded to [^125^I]ISAS and [^125^I]IPPI binding in Figure 5B,C. Competition of [^125^I]ISAS in the presence of 10 μM IPPI reduced the GM/WM ratio to 1.45, while for [^125^I]IPPI in the presence of 10 μM ISAS, the GM/WM ratio was reduced to 0.99. This confirms that [^125^I]ISAS and [^125^I]IPPI bind to similar Tau sites in the AD brain.

**Figure 6:**
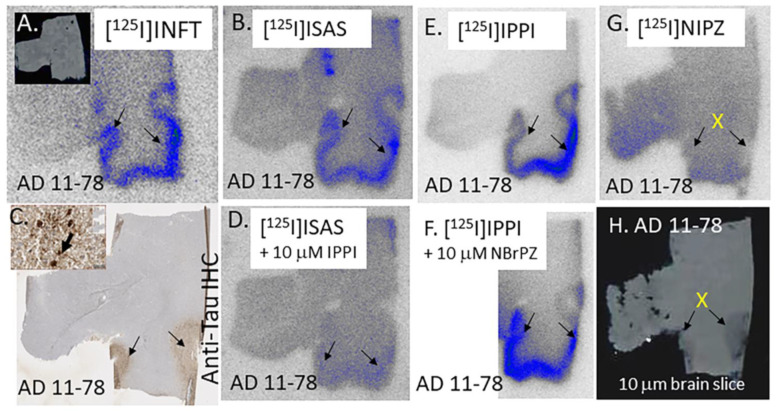
Comparison of [^125^I]Tau radiotracers: (**A**) [^125^I]INFT binding (arrows) in Tau-rich regions of human postmortem AD 11-78 brain anterior cingulate (10 μm thick sections; shown in inset). (**B**) Binding of [^125^I]ISAS to Tau (shown by arrows) in the gray matter regions of the anterior cingulate of AD 11-78 brain slice. (**C**) Anti-Tau staining of AD 11-78 showing the presence of Tau in GM regions. Inset shows magnification (20 μm) of GM region shows Tau (arrow). (**D**) Competition of [^125^I]ISAS to Tau (shown by arrows) by 10 μM IPPI in the gray matter regions of the anterior cingulate of AD 11-78 brain slice. (**E**) Binding of [^125^I]IPPI to Tau (shown by arrows) in the gray matter regions of the anterior cingulate of AD 11-78 brain slice. (**F**) No displacement of [^125^I]IPPI from Tau binding sites by 10 μM NBrPZ. (**G**) No specific binding (marked by X) of [^125^I]NIPZ to Tau regions of AD 11-78 in the gray matter regions (**H**) occurred.

**Figure 7:**
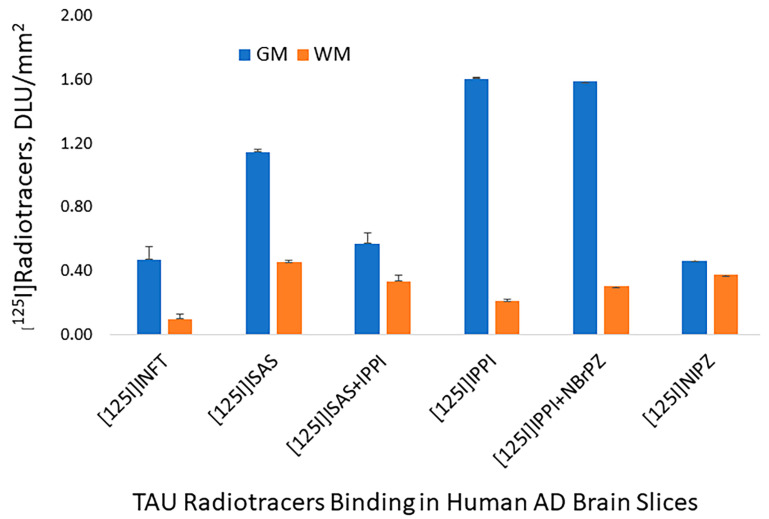
Plot showing gray matter (GM) and white matter (WM) binding of four Tau radiotracers to postmortem human AD brain slice (AD subject 11-78; Figure 6). Ratios of GM/WM were as follows: [^125^I]INFT = 4.86; [^125^I]ISAS = 3.62; [^125^I]ISAS + 10 μM IPPI = 1.70; [^125^I]IPPI = 7.74; [^125^I]IPPI + 10 μM NBrPZ = 5.26; [^125^I]NIPZ = 1.24.

In vitro binding of four radiotracers (Figure 1), [^125^I]INFT, [^125^I]ISAS, [^125^I]IPPI, and [^125^I]NIPZ were evaluated in AD 11-78 brain slices. The brain slice (Figure 6A, inset) consisted of the anterior cingulate (GM) and corpus callosum (WM). Significant [^125^I]INFT binding was observed in the GM, including the anterior cingulate (arrows in Figure 6A), while the WM consisting of corpus callosum had lower binding. A similar binding profile was observed with [^125^I]ISAS in the GM and WM (arrows in Figure 6B). The binding of [^125^I]INFT and [^125^I]ISAS correlated very well with the IHC findings of total Tau, as seen in Figure 6C (arrows and inset), confirming the binding of [^125^I]INFT and [^125^I]ISAS to regions with Tau in the brain slice. The highest levels of binding to the GM of AD 11-78 were observed with [^125^I]IPPI, as seen in Figure 6E, while little or no specific binding in the GM was seen with [^125^I]NIPZ (Figure 6G). The corresponding brain slice of the [^125^I]NIPZ experiment is seen in Figure 6H, confirming the presence of GM regions. In order to further ascertain the inability of [^125^I]NIPZ to bind to Tau sites, a competition experiment involving BrNIPZ (10 μM **10**) with [^125^I]IPPI confirmed the lack of displacement from Tau sites (Figure 6F). Thus, the rank order of binding of the four radiotracers to Tau was found to be [^125^I]IPPI> [^125^I]INFT> [^125^I]ISAS >>> [^125^I]NIPZ. A plot of GM/WM ratios of the four radiotracers is shown in Figure 7. Ratios of GM/WM were as follows: [^125^I]IPPI = 7.74 > [^125^I]INFT = 4.86 > [^125^I]ISAS = 3.62 >> [^125^I]NIPZ = 1.24. While IPPI was able to significantly reduce the binding of [^125^I]ISAS ([^125^I]ISAS + 10 μM IPPI = 1.70) suggesting similar binding sites, the pyrazine derivative NBrPZ only had a marginal effect on [^125^I]IPPI binding ([^125^I]IPPI + 10 μM NBrPZ = 5.26), suggesting a lack of binding to Tau of the pyrazine derivative.

## 3. Discussion

There is continued interest in the development of optimal Tau imaging agents to address potential shortcomings of existing ones and develop new agents for other Tauopathies [27,28,29]. The availability of such unique imaging agents will enable studies related to neurotransmitter anomalies in the AD brain [30,31]. Advancements in the antibody treatment approach to AD are also likely to gain from small molecule imaging of AD neuropathologies [32]. To further optimize the binding properties (affinity and lipophilicity) of our previously reported radioiodinated Tau imaging agents, [^125^I]IPPI and [^125^I]INFT, we investigated modifications to the pyridine ring. Two modifications were carried out. First, the iodine atom was moved from the 4-position to the 5-position in the pyridine ring. Since the Tau binding sites can accommodate the large isoquinoline ring, this change was considered not to be harmful for binding to Tau sites [20,33]. The binding of the new ISAS was comparable to INFT as shown in the Chimera–AutoDock models in Figure 2, with the only difference being in the directionality of the iodine atom. Table 1 shows the binding energies of INFT and ISAS to be very similar for the four Tau binding sites. The binding energies for IPPI were, however, lower than both INFT and ISAS. Based on this evaluation, we anticipated similar binding properties of INFT and ISAS with approximately similar lipophilicities (ISAS had a ~7% higher clogP compared to INFT, Table 1). In our previous study, compared to INFT, two other derivatives, FNFT (fluorine instead of iodine) and ClNFT (chlorine instead of iodine) were found to have reduced clogP values of 2.05 and 2.56, respectively. However, the binding affinity of these two derivatives for the Tau sites was significantly weaker by an order of magnitude compared to INFT [26]. Thus, our second alternative approach reported here was to reduce the lipophilicity of ISAS by replacing the pyridine ring with a pyrazine ring to provide NIPZ (Figure 1, **4**). Chimera–AutoDock studies with NIPZ suggested that binding to Tau would be weaker at the four sites. Molecular models suggest that the iodopyrazine ring is non-coplanar with the azaindole ring (rotation of the iodopyrazine by ~90°, Figure 2F) unlike INFT and ISAS where the iodopyridine ring is co-planar with the azaindole ring (Figure 2D,E).

Based on the structural features of the limited series of compounds, it may be surmised that the iodopyridine ring in INFT and ISAS binds in a hydrophobic pocket. The larger iodine atom enhances hydrophobic interactions and replacing the iodine with more “electronegative” substituents (such as fluorine, chlorine, or the additional ring nitrogen at the 4-position) becomes detrimental to Tau binding.

**Figure 8:**
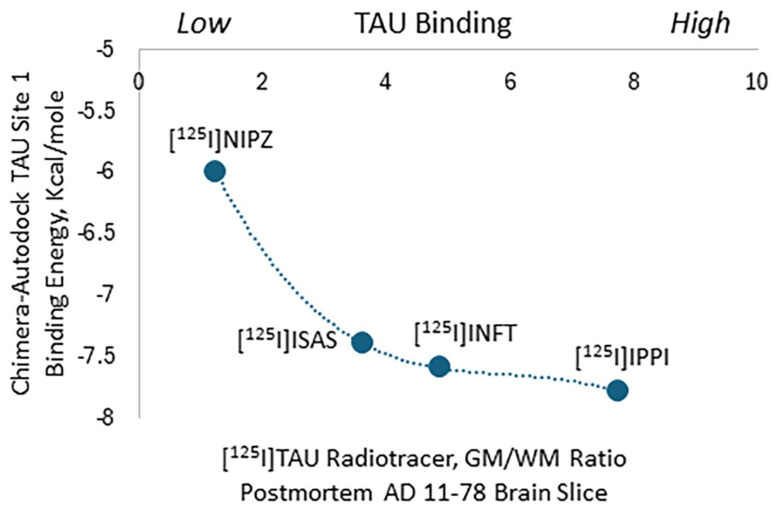
Plot of TAU binding site 1 energies measured using Chimera–AutoDock versus gray matter (GM) and white matter (WM) binding ratio of four Tau radiotracers ([^125^I]IPPI, [^125^I]INFT, [^125^I]ISAS, [^125^I]NIPZ) to postmortem human AD brain slice (AD subject 11-78).

Both [^125^I]ISAS and [^125^I]NIPZ were efficiently prepared using our previously reported methods and used for the in vitro studies [20,26]. Similar to our previous work with [^125^I]IPPI using postmortem anterior cingulate [20,25], [^125^I]ISAS exhibited selective binding of Tau-rich brain regions. Although [^125^I]ISAS has lower lipophilicity compared to [^125^I]IPPI, the higher [^125^I]IPPI GM/WM ratio compared to [^125^I]ISAS was likely related to the higher affinity of IPPI for the Tau binding sites. Mutual competition of IPPI and ISAS supported binding to similar Tau sites in the postmortem AD brain. The binding of [^125^I]INFT and [^125^I]ISAS correlated very well with IHC findings of total Tau, confirming the binding of [^125^I]INFT and [^125^I]ISAS to regions with Tau in the brain slice. Little or no specific binding in the GM was seen with [^125^I]NIPZ. This finding appeared to be consistent with our molecular modeling findings of NIPZ. Thus, the rank order of binding of the four radiotracers to Tau based on ratios of GM/WM as well as binding energies measured by Chimera–AutoDock were from high to low, [^125^I]IPPI > [^125^I]INFT > [^125^I]ISAS >> [^125^I]NIPZ (Figure 8).

In summary, less lipophilic Tau imaging agents were designed using molecular modeling using Chimera–AutoDock and subsequently radiolabeled and evaluated using in vitro postmortem AD brain slices. This resulted in a new promising Tau imaging agent, [^125^I]ISAS, which is a close analog of our previously reported [^125^I]INFT. The predictive value of Chimera–AutoDock for structurally related compounds (the same class of compounds) in terms of binding to the Tau binding sites (measured as binding energy) was good. This was ascertained for four Tau radioiodinated imaging agents. Such approaches may be useful in the development and optimization of radioiodinated imaging agents for in vivo use in AD transgenic mice models [34]. 

## 4. Materials and Methods

### 4.1. General Methods

General methods were similar to those described previously [26]. 

### 4.2. Synthesis 

5-Iodo-2-(1H-pyrrolo[2,3-c]pyridine-1-yl)pyridine (ISAS), **7**: 6-Azaindole, Figure 3, **5** (102 mg, 0.86 mmol) was treated with sodium tert-butoxide (99 mg, 1.03 mmol) in dimethylformamide (DMF, 1 mL) for 15 min at 100 °C. Subsequently, 2-chloro-4-iodopyridine, Figure 3, **6** (331 mg, 1 mmol) was added to the reaction mixture and this mixture was then heated at 100 °C for 24 h. The mixture was then cooled, 10 mL water was added, and organics were extracted using dichloromethane (CH_2_Cl_2_). The CH_2_Cl_2_ layer was dried with anhydrous magnesium sulfate and purified using preparative TLC (hexane:ethyl acetate 1:1) to provide ISAS, as an off-white solid **7** (60 mg, 0.2 mmol) in 20% yield. ISAS: mass spectra (ESI): 322 [M + H]^+^ 100%; NMR (500 MHz, CDCl_3_): δ 9.63 (s, 1H), 8.79 (d, J = 5.3 Hz, 1H), 8.38 (d, J = 5.3 Hz, 1H), 8.22 (d, J = 5.3 Hz, 1H), 7.83 (s, 1H), 7.59 (d, J = 3.45 Hz, 1H), 7.33 (d, J = 4.5 Hz, 1H), 6.75 (d, J = 3.4 Hz, 1H). 

5-Bromo-2-(1H-pyrrolo[2,3-c]pyridine-1-yl)pyrazine (NBrPZ), **10**: 6-Azaindole, Figure 3, **5** (102 mg, 0.86 mmol) was treated with sodium tert-butoxide (99 mg, 1.03 mmol) in dimethylformamide (DMF, 1 mL) for 15 min at 100 °C. Subsequently, 2,5-dibromopyrazine, Figure 4, **9** (237 mg, 1 mmol) was added to the reaction mixture and then heated at 100 °C for 24 h. The mixture was processed as described above. The CH_2_Cl_2_ layer was dried with anhydrous magnesium sulfate and purified using preparative TLC (hexane:ethyl acetate 1:1) to provide ISAS, as an off-white solid **10** (70 mg, 0.25 mmol) in 30% yield. ISAS: mass spectra (ESI): 275, 277 [M + H]^+^ 100%; NMR (500 MHz, CDCl_3_): δ 9.62 (s, 1H), 8.65 (d, J = 5.3 Hz, 1H), 8.38 (d, J = 5.3 Hz, 1H), 7.83 (s, 1H), 7.58 (d, J = 3.45 Hz, 1H), 7.42 (d, J = 4.5 Hz, 1H), 6.75 (d, J = 3.4 Hz, 1H). 

### 4.3. Molecular Modeling

Using cryo-EM structures of tau filaments from AD (PDB 503L [33]), molecular modeling methods for ISAS and NIPZ were similar to those described previously for IPPI and INFT [20,26]. 

### 4.4. Radiosynthesis 

5-[^125^I]Iodo-2-(1H-pyrrolo[2,3-c]pyridine-1-yl)pyridine, 3 [^125^I]ISAS and 5-[^125^I]Iodo-2-(1H-pyrrolo[2,3-c]pyridine-1-yl)pyridine, 3 [^125^I]NIPZ: To a solution of ISAS, **7** (10 mg; 31 μmol) or NBrPZ, **10** (10 mg; 36 μmol) in anhydrous triethylamine (1 mL) under nitrogen, bis(tributyltin) (60 mg; 103 μmol) and Tetrakis(triphenylphosphine)palladium(0) (5 mg; 4.3 μmol) were added. This reaction mixture was refluxed overnight at 90 °C. The dark yellow crude reaction mixture was purified over a prep silica gel TLC plate using hexane:ethyl acetate 1:1 as a solvent. The product was isolated as an oil (Figure 3, **8** and Figure 4, **11**) in 40% yield. Mass spectra (ESI) for **8**: 482 (55%), 484 (100%), 486 (87%); [M + H]^+^; NMR (500 MHz, CDCl_3_): δ 9.63 (s, 1H), 8.87 (d, J = 5.3 Hz, 1H), 8.38 (d, J = 5.3 Hz, 1H), 8.22 (d, J = 5.3 Hz, 1H), 7.79 (s, 1H), 7.59 (d, J = 3.45 Hz, 1H), 7.33 (d, J = 4.5 Hz, 1H), 6.75 (d, J = 3.4 Hz, 1H), 1.61 (m, 6H), 1.38 (m, 6H), 1.19 (m, 6H), 0.91 (t, 9H).

For **9**: 483 (43%), 485 (100%), 487 (77%). NMR (500 MHz, CDCl_3_): δ 9.62 (s, 1H), 8.63 (d, J = 5.3 Hz, 1H), 8.34 (d, J = 5.3 Hz, 1H), 7.80 (s, 1H), 7.58 (d, J = 3.45 Hz, 1H), 7.42 (d, J = 4.5 Hz, 1H), 6.75 (d, J = 3.4 Hz, 1H), 1.63 (m, 6H), 1.38 (m, 6H), 1.19 (m, 6H), 0.92 (t, 9H).

Iodine-125 sodium iodide was purchased from American Radiolabeled Chemicals, Inc., St. Louis, MO, USA (iodine-125 sodium iodide, carrier-free (specific activity = 643 MBq/μg) in 0.01 N NaOH). A radioiodination hood (CBS Scientific, Inc., San Diego, USA) placed inside a fume hood designated to handle radioactive materials was used to carry out iodine-125 radiolabeling of **8** and **11** using our previously reported methods [20,26]. Purified ethanolic solutions of [^125^I]ISAS, **3** and [^125^I]NIPZ, **4** at concentrations of approximately 1 MBq/mL were used for in vitro experiments. Both [^125^I]ISAS, **3** and [^125^I]NIPZ, **4** were monitored by RadioTLC and found to be stable over several months when stored at −20 °C.

### 4.5. Human Tissue

All postmortem human brain studies were approved by the Institutional Biosafety Committee of the University of California, Irvine. Human postmortem brain tissue samples were obtained from Banner Sun Health Research Institute, Sun City, AZ, USA, a brain tissue repository.

### 4.6. In Vitro Postmortem Human Brain Autoradiography

Human anterior cingulate sections containing corpus callosum were sectioned from the AD subjects. Our previously reported procedures for [^125^I]INFT and [^125^I]IPPI were used for the binding studies [20,26]. Brain sections were air dried, exposed overnight on a phosphor film, and then placed on the Phosphor Autoradiographic Imaging System/Cyclone Storage Phosphor System (Packard Instruments Co., Booton, NJ, USA). Regions of interest (ROIs) were drawn on the slices and the extent of binding of [^125^I]IPPI was measured in DLU/mm^2^ using the OptiQuant acquisition and analysis program (Packard Instruments Co.). Statistical differences between groups were determined using Microsoft Excel 16. Statistical power was determined using the Student’s *t* test and *p* < 0.05 was considered to be significant.

### 4.7. Immunohistochemistry

University of California–Irvine, Pathology Services, used Ventana BenchMark Ultra protocols for immunostaining of brain sections using procedures described previously [20,26].

## Figures and Tables

**Table 1:** Binding affinity, lipophilicity, and binding energies of Tau agents.

Compound #	Name	^a^ Tau Affinity, IC_50_	^b^ cLogP	^c^ Molecular Modeling Site 1, 2, 3, 4 (Kcal/mol)	Reference
1	IPPI	2.14 × 10^−8^	4.34	−7.8, −8.1, −8.2, −7.5	[20]
2	INFT	7.32 × 10^−8^	2.96	−7.6, −6.6, −6.6, −6.2	[26]
3	ISAS	4.69 × 10^−8^	3.18	−7.4, −6.7, −6.9, −6.4	This work
4	NIPZ	>10^−6^	2.45	−6.0, −6.3, −6.4, −5.9	This work
10	NBrPZ	>10^−6^	2.18	−6.8, −6.2, −6.5, −6.1	This work

^a^ Binding affinities were measured using [^125^I]IPPI in postmortem AD brain slices, autoradiographically; ^b^ logP was calculated using methodology developed by Molinspiration; ^c^ Binding energies were calculated using Chimera–AutoDock procedures described in [20].

## Data Availability

The data that support the findings of this study are available from the corresponding author upon reasonable request.
